# Functional reorganization of brain networks from childhood to adolescence: A multi-level approach

**DOI:** 10.1016/j.ijchp.2026.100700

**Published:** 2026-06-17

**Authors:** Merida Galilea Tapia-Medina, Raquel Cosío-Guirado, Maribel Peró-Cebollero, Cristina Cañete-Massé, Erwin Rogelio Villuendas-González, Joan Guàrdia-Olmos

**Affiliations:** a Department of Social Psychology and Quantitative Psychology, University of Barcelona, Barcelona, Spain; b Institute of Complex Systems, University of Barcelona, Barcelona, Spain; c Institute of Neuroscience, University of Barcelona, Barcelona, Spain; d Faculty of Psychology, Universidad Michoacana de San Nicolás de Hidalgo Morelia, Mexico

**Keywords:** Degree centrality, Seed-based functional connectivity, Brain networks, Resting-state fMRI, Neurodevelopment

## Abstract

**Background/objective:**

During childhood and adolescence, functional connectivity undergoes distinct age-related maturational trajectories within and between networks. However, normative connectivity patterns across development remain insufficiently characterized.

**Methods:**

This study integrates voxel-wise degree centrality, degree centrality-derived seed-based connectivity, and network-level analyses to examine large-scale functional organization in a large multisite sample of 322 typically developing children (7–10.99 years) and adolescents (11–15 years) from the Autism Brain Imaging Data Exchange I and II repositories.

**Results:**

Across all analytical levels, adolescents exhibited strengthened functional connectivity of subcortical hubs -the thalamus and the basal ganglia- with the Ventral Attention Network and Frontoparietal Network, emerging as the key hubs of network integration in adolescence. Children, by contrast, showed stronger functional connectivity within posterior sensory-perceptual nodes, particularly involving angular and occipital regions.

**Conclusions:**

Our findings delineate a developmental reorganization from sensory-anchored functional architectures in childhood toward increasingly integrated subcortical, attentional and frontoparietal systems in adolescence, supporting emerging capacities for cognitive-control and goal-directed behavior. This multilevel characterization offers a normative reference framework for interpreting variability in neurodevelopment and, ultimately, for identifying potential early deviations.

## Introduction

Nearly two-thirds of neuropsychiatric disorders emerge during the first two decades of life (Wainberg et al., 2022), highlighting childhood and adolescence as critical periods for brain maturation and vulnerability. Early childhood is characterized by rapid maturation of sensory, motor, and regulatory functions and coincides with the onset of disorders related to impulse control, anxiety, and the autism spectrum (Kessler et al., 2005; Uddin et al., 2025). Adolescence represents a second major developmental transition marked by cognitive, affective, and social changes that support increasing independence and continued maturation of executive functions such as response inhibition, working memory, and planning (Iorfino et al., 2019). Deviations from typical developmental trajectories, shaped by socioemotional and environmental factors, are thought to contribute to prolonged vulnerability to psychiatric disorders (Tervo-Clemmens et al., 2023). Accordingly, most mental illnesses are now conceptualized as neurodevelopmental in nature, arising from disruptions in typical brain maturation (Mulraney et al., 2021). Despite extensive research on clinical populations (Zhang et al., 2021), normative neurodevelopment remains comparatively understudied. Characterizing typical developmental trajectories is essential for interpreting interindividual variability, informing models of cognitive and behavioral maturation, and identifying sensitive periods relevant for prevention and intervention (Mills et al., 2021; Ouyang et al., 2024). Studying development therefore requires approaches capable of capturing both structural and functional changes across time.

Resting-state functional magnetic resonance imaging (rs-fMRI) has become a cornerstone technique for investigating human brain development (Li et al., 2019; Welvaert & Rosseel, 2014). By measuring spontaneous fluctuations in the blood oxygen level-dependent (BOLD) signal, rs-fMRI reveals intrinsic brain activity and functional connectivity in the absence of external demands (Biswal et al., 1995; Fox & Raichle, 2007; Razi & Friston, 2016). This approach is particularly suitable for pediatric populations, as it minimizes task-related variability and participant burden while enabling standardized acquisition across individuals (Bernal, 2022; Whitfield-Gabrieli et al., 2020). Moreover, rs-fMRI captures intrinsic functional systems such as default mode and executive control networks, making it well suited for mapping normative developmental trajectories and identifying early deviations associated with neuropsychiatric risk.

Beyond global functional connectivity measures, graph-theoretical approaches conceptualize the brain as a complex network and provide complementary insights into its functional organization (Sporns et al., 2005). Within this framework, degree centrality (DC) quantifies the number and strength of direct functional connections between a voxel and the rest of the brain, reflecting its importance within the whole-brain network (Buckner et al., 2009; Chen et al., 2024; Zuo et al., 2012). As a fully data-driven voxel-wise metric, DC enables unbiased exploration of global connectivity patterns without requiring predefined regions of interest (Telesford et al., 2011; Cañete-Massé et al., 2023; Shan et al., 2023).

DC has been widely used to identify alterations in resting-state functional networks across psychiatric and neurological disorders, including depression (Zhang et al., 2022), schizophrenia (Chen et al., 2015), substance abuse (Su et al., 2025), autism (Zhou et al., 2024), attention-deficit/hyperactivity disorder (Zhou et al., 2019), early-onset schizophrenia (Cheng et al., 2025), epilepsy (Ran et al., 2025), and depression in youth (Mo et al., 2025). These findings highlight DC as a sensitive marker of network-level dysfunction. However, despite its growing clinical application, DC remains scarcely studied in healthy children and adolescents (Tapia-Medina et al., 2025b), limiting its use for characterizing normative developmental trajectories. In parallel with voxel-wise metrics, network-based approaches examine the global organization and integration of functional systems across development (van den Heuvel & Hulshoff Pol, 2010). Understanding how large-scale networks mature in healthy populations is essential for establishing normative benchmarks and identifying early deviations linked to vulnerability for mental disorders (Chai et al., 2017; Grayson & Fair, 2017). Prior work has examined typical development using graph-theoretical measures such as eigenvector centrality (Sato et al., 2015; Kolskår et al., 2018), demonstrating age-related reorganization of key network nodes.

While network-level measures characterize global architecture, seed-based functional connectivity remains one of the most widely used rs-fMRI approaches for examining interactions between specific regions and the rest of the brain Catalino et al. (2020). Seed-based analyses allow targeted investigation of systems supporting cognitive and socioemotional functions but typically rely on predefined regions of interest. DC-derived seed-based approaches remain rare in pediatric populations, with few exceptions (Xiao et al., 2016; Fan et al., 2021; Tian et al., 2023; Zhou et al., 2019), despite their potential to provide data-driven and developmentally sensitive seed selection. Graph-theoretical network construction methods further enable exploration of large-scale functional organization and integration across development (van den Heuvel & Hulshoff Pol, 2010). Applying these approaches to healthy pediatric populations helps characterize maturation of systems such as default mode and executive control networks, providing essential benchmarks for normative development (Huang et al., 2021; Schielen et al., 2025).

Within this framework, we acknowledge that extensive prior research has characterized developmental shifts in functional segregation and integration (Fair et al., 2007; Uddin, 2010), mapped trajectories of network organization (Marek et al., 2015; Sun et al., 2025), and synthesized lifespan connectivity changes (Cosío-Guirado et al., 2024; Edde et al., 2021). However, these studies typically examine connectivity at a single analytical scale or rely on a priori selection of regions. Consequently, a critical gap remains in understanding how specific maturational changes in local hubs (voxel-level) cascade into global network reorganization (edge-level) within the same cohort. To properly distinguish pathological deviations, often subtle and diffuse in neurodevelopmental conditions, from typical maturational plasticity, a strictly harmonized, multi-level reference framework is required.

To properly investigate this, we applied a three-step hierarchical, fully data-driven pipeline that integrates voxel-wise DC, seed-based FC, and network construction. This framework has two primary aims. First, to identify resting-state connectivity patterns that best characterize typical neurodevelopment. Second, to examine age-related differences in network FC by comparing children and adolescents. We applied this framework to a large sample of typically developing participants aged 7–15 years from the Autism Brain Imaging Data Exchange (ABIDE I and ABIDE II) datasets. This is the first study to integrate seed-based and network construction approaches in a sample of typically developing children and adolescents.

## Methods

### Participants

We used the available ABIDE (http://fcon_1000.projects.nitrc.org/indi/abide/) dataset for the present study. The ABIDE initiative includes two collections: ABIDE I (Di Martino et al., 2013) and ABIDE II (Di Martino et al., 2017) datasets that aggregate rs-fMRI and anatomical MRI data from multiple research sites. It was designed to facilitate the study of brain connectivity in individuals with autism spectrum disorder (ASD) and typically developing (TD) controls. In accordance with the ethics board policies, our study was exempt from ethical review.

The sample consisted of typically developing (TD) participants from the ABIDE I and II datasets. To enhance the robustness of subsequent statistical analyses, only datasets meeting the following criteria were included: (1) participants aged 7 to 15 years, (2) reported IQ assessment with a standardized score >80, (3) a resting-state fMRI protocol in which participants were awake with eyes open, fixed on a cross, and (4) a minimum sample size of 20 participants per dataset. In this study we included 10 collections sites (ABIDEI-KKI, ABIDEI-SDSU, AIBDEI-UCLA_1, ABIDEI-UM_1, ABIDEI-UM_2, ABIDEII-KKI_1, ABIDEII-NYU_1, ABIDEII-OHSU_1, ABIDEII-SDSU_1 ABIDEII-TCD_1). This initial sample consisted of 372 TD children and adolescents (116 females, 256 males; M = 10.8 ± 1.8 years).

Following the initial selection, a two-step quality control (QC) procedure was conducted. First, all images were visually inspected using the DPABI Quality Control Tool (https://rfmri.org/DPABI; Yan et al., 2016) to identify participants with severe head motion in the T1-weighted image, poor functional image coverage, or misregistration between structural and functional images. Due to this visual inspection control, 39 participants were excluded. Second, as pediatric population can present excessive movement, mean head motion was quantified using Jenkinson’s framewise displacement (FD; Jenkinson et al., 2002). Following the criteria of Smith et al. (2022) and Song et al. (2024) for high-movement populations, we removed participants exceeding mean FD 0.3 mm. As a result, 11 participants were excluded due to excessive head motion. After the entire QC, the final sample comprised 322 TD participants aged 7–15 years (101 females, 221 males; M = 10.9 ± 1.2 years). This sample was divided into two discrete age groups: a children's group (7–10.99 years, n = 169; 62 females, 107 males; M = 9.5 ± 0.8 years) and an adolescents' group (11–14.99 years, n = 153; 39 females, 114 males; M = 12.5 ± 1.8 years).

The age intervals were chosen to capture subtle developmental transitions across relatively narrow age spans, which is particularly relevant in the context of childhood and adolescence, where brain maturation occurs rapidly and non-linearly. This categorization is consistent with prior neurodevelopmental research and aligns with the recommendations of Sala-Llonch et al. (2015) and Xu et al. (2021), who advocate for fine-grained age groupings to enhance sensitivity to developmental changes in functional brain architecture, maximize age resolution while ensuring statistical power by keeping at least ten subjects for each group. Recent studies have adopted comparable strategies, defining short and neurodevelopmentally informed age bins to capture age-related effects more precisely (Içer, 2019; Tian et al., 2023). In line with recent meta-analytic findings (Tapia-Medina et al., 2025b), such divisions enhance both the sensitivity and interpretability of group-level comparisons in developmental functional imaging.

### fMRI data pre-processing

Image preprocessing was conducted using the Data Processing Assistant for Resting-State fMRI (DPARSF 5.5 Advanced Edition; Yan & Zang, 2010; Yan et al., 2016; http://rfmri.org/DPARSF). This pipeline, implemented in MATLAB, is based on SPM12 (http://www.fil.ion.ucl.ac.uk/spm) and DPABI. Initially, the first ten functional volumes were discarded to minimize potential effects related to participants adapting to the scanner and to allow for proper magnetization equilibrium. The remaining functional images underwent slice-timing correction based on their acquisition times, followed by head motion estimation. Nuisance signals, including white matter and cerebrospinal fluid fluctuations, linear trends, and the 24 Friston head-motion parameters (Friston et al., 1996), were regressed out. This 24-parameter model has been shown to successfully reduce the influence of motion effects better than other models (Nomi & Uddin, 2015; Satterthwaite et al., 2012; Yan et al., 2013). Subsequently, functional images were co-registered to their respective structural images, which were then segmented and normalized to Montreal Neurological Institute (MNI) 152 space using Diffeomorphic Anatomical Registration Through Exponentiated Lie (DARTEL) (Ashburner, 2007). Functional images were also normalized to MNI space via the computed warping parameters and resampled to 3 mm isotropic voxels. To improve inter-subject alignment, anatomical images were normalized to MNI space using the DARTEL algorithm implemented in DPABI. This method has been widely used in large-scale developmental neuroimaging studies and has demonstrated high registration accuracy across diverse age ranges, including children and adolescents (Guo et al., 2019; Ingalhalikar et al., 2021; Kim et al., 2021; Ma et al., 2026; Ruan et al., 2024).

### Estimation of DC

DC analysis were performed by using the DPABI/DPARSF. Based on the preprocessed and unsmoothed fMRI data, voxel-wise Pearson correlation coefficients were computed between each voxel and all other voxels in the brain to generate the whole-brain FC matrix (Buckner et al., 2009; Zuo et al., 2012). To reduce spurious correlations, a threshold of *r* > 0.25 was applied (Buckner et al., 2009; Chen et al., 2024; Su et al., 2025). Weighted DC values were then obtained by summing the correlation coefficients of suprathreshold connections for each voxel. Then, the resulting individual DC maps were normalized using Fisher’s r-to-z transformation and spatially smoothed with a 4 mm full-width at half-maximum (FWHM) Gaussian kernel to improve signal-to-noise ratio and spatial consistency across participants.

These smoothed z-DC maps were harmonized through DPABI Harmonization (Wang et al., 2023, 2024) to minimize the potential biases and non-biological variability induced by site and scanner effects. The selected method was ComBat (Fortin et al., 2017, 2018; Johnson et al., 2007)/CovBat (Chen, Beer et al., 2021) through a parametric approach without covariate adjustment. We set the principal component analysis (PCA) covariance threshold to 90%.

Once harmonized, these z-DC maps were used for a *t*-test comparison between the children’s and adolescents’ groups to identify the initial seeds. Correction for multiple comparisons was performed using Gaussian Random Field (GRF) theory, following the recommendations by Eklund et al. (2016), with a voxel-level threshold of p < 0.001 and a cluster-level threshold of p < 0.05. To ensure that the detected clusters reflected not only statistical significance but also meaningful effect sizes, only regions showing an estimated r ≥ 0.20 were retained for further seed-based analyses. Marek et al. (2022) introduced magnitude-based thresholds to address the typically small effect sizes in brain-behavior associations. Similarly, this filtering paradigm is broadly crucial in neuroimaging to mitigate inflated effects and ensure reproducibility (Poldrack et al., 2017). Consistent with this, Gignac and Szodorai (2016) noted that r = 0.20 represents a moderate effect size in individual differences research. Therefore, we applied this criterion of r ≥ 0.20 as a conservative heuristic to identify stable maturational patterns. Accordingly, peak t values were converted to r values, and only clusters meeting this minimum effect size threshold were retained (Cohen, 1988).

### Seed-based FC

As the second stage of our multi-level pipeline, seed-based FC analyses were conducted to map the FC originating from the previously identified developmental hubs using DPABI/DPARSF. Regions showing significant group differences in DC (voxel-level *p* < 0.001, cluster-level *p* < 0.05, GRF corrected, and *r* ≥ 0.20, a threshold chosen to ensure effect sizes of sufficient magnitude and robustness, as detailed before) were used as seeds. To maintain biological specificity and minimize Type I error, this expansion was limited to a single iteration, preserving the analytical hierarchy. Each seed was defined as a 6-mm-radius sphere centered at the peak voxel of the corresponding cluster identified in the DC analysis. For each participant, the mean time series within each seed was extracted and correlated with the time series of all other voxels in the brain to generate voxel-wise FC maps. The resulting correlation coefficients were then converted to z-values using Fisher’s r-to-z transformation, yielding the z-FC maps used for subsequent group-level analysis.

Between-group comparisons of the *z*-FC maps were performed using two-sample *t*-test using the criteria of PALM (Permutation Analysis of Linear Models) with TFCE (Threshold-Free Cluster Enhancement) correction, which reaches the best balance between family-wise error and test-retest reliability (Chen et al., 2018; Winkler et al., 2016). A total of 5000 permutations were performed, and the cluster *p*-value was set to *p* < 0.05. Again, we further applied the r ≥ 0.20 effect-size threshold to retain only clusters showing meaningful associations. Four *t*-tests were conducted, one for each of the four initial seeds derived from the DC analysis, yielding 25 additional significant ROIs. In total, 29 ROIs were identified. One of the 29 ROIs was excluded from the statistical analyses because its spatial location fell partially outside the brain volume, projecting into the brainstem. Similar procedures have been reported in previous neuroimaging studies that removed ROIs located in peripheral or non‐brain regions to avoid contamination from cerebrospinal fluid or misalignment artifacts (Cao et al., 2025; Islam et al., 2024; van Lutterveld et al., 2022). As a result, 28 ROIs were considered for the network construction.

### Network-level connectivity

ROI-wise time series were extracted in DPABI/DPARSF using 28 spherical ROIs (6-mm radius) centered at the MNI coordinates identified previously (four from DC and 25 from seed-based FC). For each participant, DPABI computed the mean BOLD signal across all voxels within each sphere, after detrending and temporal filtering according to the preprocessing parameters. The resulting time series represented the average activity of each ROI. Then, in DPABINet 1.3 module (Yan et al., 2024), Pearson’s correlation coefficients were then computed between all extracted ROI time series to construct individual FC matrices. The resulting correlation coefficients were converted to Fisher’s z-values to improve normality, yielding the ROI-to-ROI connectivity matrices used for network construction.

To better describe significant clusters obtained in network-based contrast, we also classified suprathreshold edges by their membership in the networks defined by Yeo et al. (2011) networks using a Matlab script, which identifies the Yeo networks using Buckner and Choi parcellations (Buckner et al., 2011; Choi et al., 2012). The seven networks are the Visual Network (VN), Somatosensory-Motor Network (SMN), Dorsal Attention Network (DAN), Ventral Attention Network (VAN), Limbic Network (LN), Frontoparietal Network (FPN), and Default Mode Network (DMN).

### Statistical analysis

Group comparisons of the ROI-to-ROI connectivity matrices were conducted using DPABINet. For each participant, individual network matrices were entered into a two-sample *t*-test comparing the children’s and adolescents’ groups. Statistical inference was performed using PALM with 5000 permutations and False Discovery Rate (FDR) correction to control for multiple comparisons across edges (Chen et al., 2018; Winkler et al., 2016). To account for potential site-related variability, site was included as a covariate in the model. Given that current implementations of ComBat/CovBat harmonization are primarily optimized for voxel-wise fMRI data (e.g., z-maps) rather than for matrix-based connectivity measures, modeling site as a covariate was considered a more robust and interpretable way to control for site effects while preserving genuine FC differences. Significant between-group differences were identified at *p* < 0.05 (FDR-corrected) for the 28 coordinates used.

Significant clusters were localized in standard MNI space, and their corresponding anatomical labels were identified using the Automated Anatomical Labeling (AAL) atlas (Tzourio-Mazoyer et al., 2002). Visualization of statistical maps was performed using DPARSF and DPABINet.

## Results

### Degree centrality

**Fig. 1:**
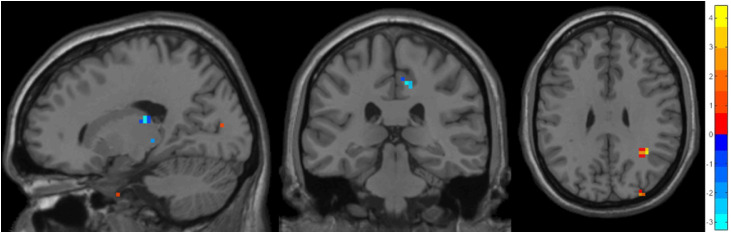
Sagittal, axial and coronal planes representation of significant between-group differences in DC.

*Note.* The color bar indicates the intensity of the difference (*t*), with yellow representing significantly increased DC in children compared with adolescents and light blue representing significantly decreased DC in children compared with adolescents. Maps were visualized using DPABI Viewer.

Group comparisons of voxel-wise DC revealed significant between-group differences in four clusters (GRF-corrected, voxel-level p < 0.001, cluster-level p < 0.05, and r ≥ 0.20). Compared with adolescents, children showed higher DC values in the right angular gyrus and the right superior occipital gyrus. Conversely, adolescents exhibited higher DC values in the right thalamus and right middle cingulum. These four peak coordinates were selected as seeds for subsequent seed-based FC analysis. Table 1 shows the significant differences between groups in DC with MNI coordinates. Fig. 1 provides a visual representation of these regions.

**Table 1:** Significant between-group differences in DC.

Comparison	AAL peak region	MNI peak coordinates	Number of voxels	*t* peak
Children > Adolescents	∼Angular R	36 −51 30	17	4.0977
	∼Occipital Sup R	30 −90 30	3	3.7346
Adolescents > Children	Thalamus R	18 −21 18	11	−3.8500
	Cingulum Mid R	9 −30 45	6	−3.8949

*Note.* AAL = Automated Anatomical Labeling atlas; MNI = Montreal Neurological Institute; ∼ = Approximately, AAL atlas area closer to the *t* peak; All results survived GRF correction for multiple comparisons (voxel-level *p* < 0.001, cluster-level *p* < 0.05) and *r* ≥ 0.20.

### Seed-based FC analysis

To investigate specific regional pathways, seed-based FC analyses were performed to map connections originating from local hubs. We used the four regions that showed significant between-group differences in degree centrality as seeds (Table 1, Fig. 1). Among them, three seeds (the right thalamus, right superior occipital gyrus, and right middle cingulum) showed significant differences between children and adolescents in voxel-wise FC maps (TFCE-corrected *p* < 0.05, *r* ≥ 0.20). No significant results were found for the right angular gyrus seed.

For the right thalamus seed, adolescents exhibited higher FC with the pallidum, putamen, caudate, inferior temporal gyrus and precentral gyrus.

Using the right superior occipital as seed, adolescents showed increased FC with the precuneus, caudate and hippocampus, whereas children displayed stronger connectivity with the thalamus.

Finally, the right middle cingulate seed revealed greater FC in adolescents with frontal and temporal areas, including the superior temporal pole, middle frontal gyrus, and supramarginal gyrus, as well as with posterior regions such as the cuneus, precuneus, and cingulum.

Details of the significant clusters for each seed are listed in Table 2.

**Table 2:** Between-group differences in seed-based functional connectivity using DC-derived seed regions.

Seed region	AAL significant region	MNI peak coordinates			*t* peak	Comparison
Right thalamus	Temporal_Inf_R	48	−45	−21	−5.3962	Children < Adolescents
	Putamen_R	21	9	−3	−5.0216	Children < Adolescents
	Pallidum_R	21	−3	3	−5.4193	Children < Adolescents
	∼Pallidum_R	12	3	3	−5.7422	Children < Adolescents
	Pallidum_L	−15	3	3	−5.2241	Children < Adolescents
	∼Pallidum_R	−24	−12	6	−5.1627	Children < Adolescents
	Putamen_L	−30	0	6	−5.065	Children < Adolescents
	∼Putamen_R	24	−9	9	−5.3707	Children < Adolescents
	∼Caudate_R	15	3	12	−5.5192	Children < Adolescents
	Precentral_L	−42	−15	57	−5.1784	Children < Adolescents
Right superior occipital gyrus	∼Precuneus_R	30	−45	6	−5.3466	Children < Adolescents
	∼Thalamus_R	3	−3	6	5.0497	Adolescents < Children
	∼Caudate_R	3	15	6	−5.4003	Children < Adolescents
	∼Hippocampus_R	24	−36	15	−5.3892	Children < Adolescents
Right middle cingulate gyrus	∼Temporal_Pole_Sup_R	54	12	−3	−5.0917	Children < Adolescents
	Cuneus_R	15	−72	30	−5.1314	Children < Adolescents
	Frontal_Mid_L	−33	33	30	−5.0462	Children < Adolescents
	Frontal_Mid_R	33	39	33	−5.5463	Children < Adolescents
	Cuneus_R	6	−75	36	−5.0241	Children < Adolescents
	SupraMarginal_R	60	−39	39	−5.385	Children < Adolescents
	Cingulum_Mid_L	−3	3	39	−5.6109	Children < Adolescents
	Cingulum_Mid_R	3	9	42	−5.5782	Children < Adolescents
	Precuneus_R	3	−45	51	−5.0113	Children < Adolescents
	Precuneus_R	3	−48	63	−5.4961	Children < Adolescents

*Note.* AAL = Automated Anatomical Labeling atlas; MNI = Montreal Neurological Institute; ∼ = Approximately, AAL atlas area closer to the *t* peak; All results survived TFCE correction for multiple comparisons (*p* < 0.05) and *r* ≥ 0.20.

### Network-level connectivity

To assess global integration across large-scale systems, network-based analyses were performed using the 28 ROIs derived from DC and seed-based FC analysis. All ROIs were classified according to the seven large-scale functional networks defined by Yeo et al. (2011) as well as an additional category (Others) including regions not assigned to any of these networks. Table 3 shows the classification.

**Table 3:** Classification of the significant regions in Yeo networks.

VN	SMN	DAN	VAN	FPN	Others
∼Occipital_Sup_R	Precentral_L	Temporal_Inf_R	Cingulum_Mid_R	∼Caudate_R	Thalamus_R
Cuneus_R	∼Temporal_Pole_Sup_R	Precuneus_R	Putamen_R	Cuneus_R	∼Angular_R
		Precuneus_R	∼Pallidum_R	SupraMarginal_R	Pallidum_R
			Putamen_L		∼Pallidum_R
			∼Putamen_R		Pallidum_L
			Frontal_Mid_L		∼Precuneus_R
			Frontal_Mid_R		∼Thalamus_R
			Cingulum_Mid_L		∼Caudate_R
			Cingulum_Mid_R		∼Hippocampus_R

*Note.* VN = Visual Network; SMN = Somatosensory-Motor Network; DAN = Dorsal Attention Network; VAN = Ventral Attention Network; FPN = Frontoparietal Network; ∼ = Approximately, AAL atlas area closer to the *r* peak.

Regarding the VN, adolescents exhibited higher FC between the cuneus and nodes of the DAN and the VAN.

Within the SMN, the left precentral gyrus and right superior temporal pole emerged as the most connected nodes, both showing higher FC in adolescents. The right superior temporal pole, in particular, displayed numerous and intense connections with multiple nodes of the VAN and some nodes that are not included in the Yeo parcellations. The left precentral gyrus also showed enhanced FC with regions in these same networks.

For the DAN, the right inferior temporal gyrus showed increased FC in adolescents with one node of the VAN and another node not included in the Yeo parcellations. The first right precuneus was more connected in adolescents with a node within the VN and another within the VAN, while the second right precuneus showed the same pattern but with additional connections to several VAN nodes.

Regarding the VAN, most of its nodes exhibited widespread and robust increases in FC in the adolescents’ group. The right middle cingulum showed higher FC with the VN, SMN, DAN, numerous nodes within the VAN, and the FPN. The putamen presented increased connectivity with the SMN, DAN, VAN, FPN, and regions not included in the Yeo networks. The right pallidum appears to be increased with nodes of the SMN, VAN, FPN, and several nodes not included in the Yeo parcellations. The left middle frontal gyrus was more connected in adolescents with a node of the SMN, a node of the DAN, nearly all VAN nodes, and a region outside the Yeo networks. The right middle frontal gyrus showed connections with VAN nodes and a single region not included in the Yeo parcellations. The left middle cingulum displayed higher FC with a node in the DAN and nodes of the VAN, whereas the right middle cingulum showed significant intra-network FC, connecting exclusively with other VAN nodes.

In the FPN, again, all ROIs seemed to be increased in the adolescents’ group. The right caudate showed increased FC in adolescents with several regions of the VAN and several others not considered in the Yeo networks. The remaining FPN regions displayed enhanced FC exclusively with the VAN.

Finally, in the Others category, the first right thalamus exhibited widespread and intense FC in adolescents with all nodes of the SMN, a node of the DAN, several nodes of the DAN, a node of the FPN and several nodes outside the Yeo parcellations. The second right thalamus showed enhanced FC in the adolescent’s group with a region not included in the Yeo networks, while showing higher FC in children with another node also outside these parcellations. The right angular gyrus presented increased FC in adolescents with some nodes not included in the Yeo networks, but conversely exhibited increased FC in the children’s group with another region outside the Yeo networks. The first right pallidum displayed higher FC in adolescents with the SMN, the VAN, the FPN and regions outside the Yeo networks. The second right pallidum and the left pallidum both showed heightened FC in adolescents with the VAN, FPN and regions beyond the Yeo parcellations. Both the right precuneus and the right caudate exhibited increased FC in adolescents with a node not included in the Yeo networks. Finally, the hippocampus showed increased FC in adolescents with a node of the VAN and a node outside the Yeo networks.

**Fig. 2:**
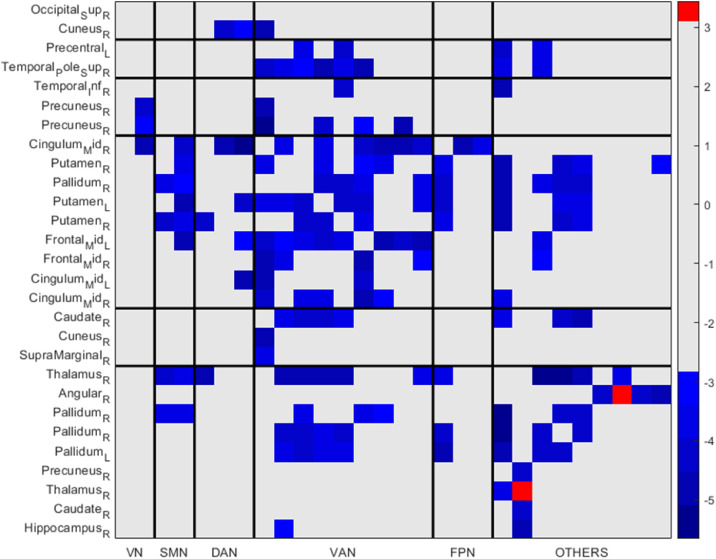
Edge plot showing between-group differences in ROI-to-ROI functional connectivity between children and adolescents.

*Note.* VN = Visual Network; SMN = Somatosensory-Motor Network; DAN = Dorsal Attention Network; VAN = Ventral Attention Network; FPN = Frontoparietal Network; Others = regions not assigned to any of Yeo networks. Blue colors show areas that have significantly increased connectivity in adolescents compared to children. Red areas show significantly increased connectivity in children compared with adolescents. The plot was generated using the DPABINet viewer with Network-Based Statistic (NBS; Zalesky et al., 2010) correction (edge-level *p* < 0.001, component-level *p* < 0.05).

**Fig. 3:**
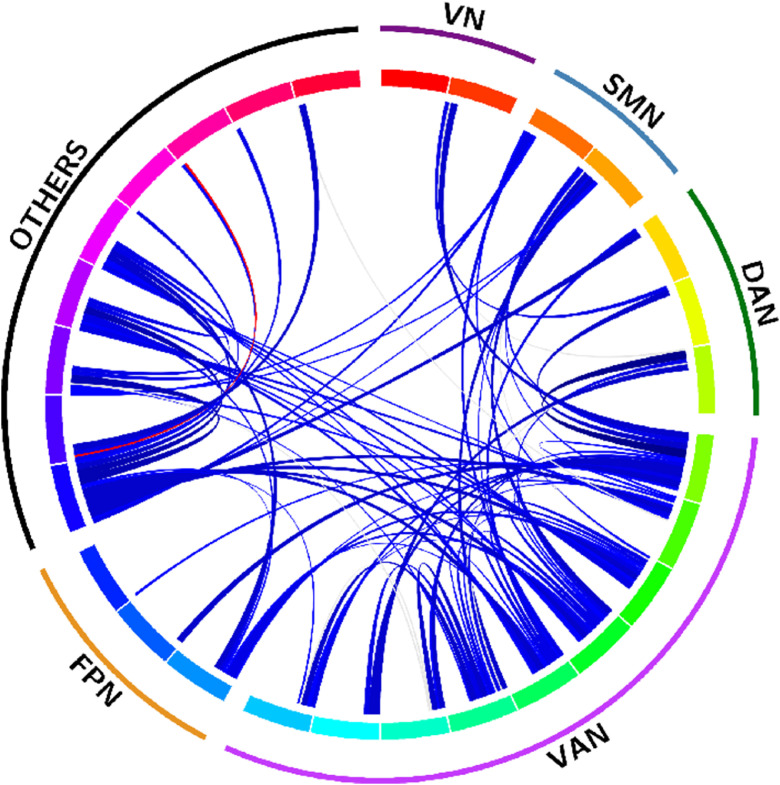
Circular connectogram illustrating between-group differences in functional connectivity between children and adolescents.

*Note.* The connectogram was generated using the Circos tool (Krzywinski et al., 2009) from results obtained with the NBS (Zalesky et al., 2010) correction in DPABINet (edge-level *p* < 0.001, component-level *p* < 0.05). Nodes are grouped according to the large-scale cortical networks defined by Yeo et al. (2011). Blue lines show areas that have significantly increased connectivity in adolescents compared to children, while red lines show areas that have increased connectivity in children compared with adolescents.

**Fig. 4:**
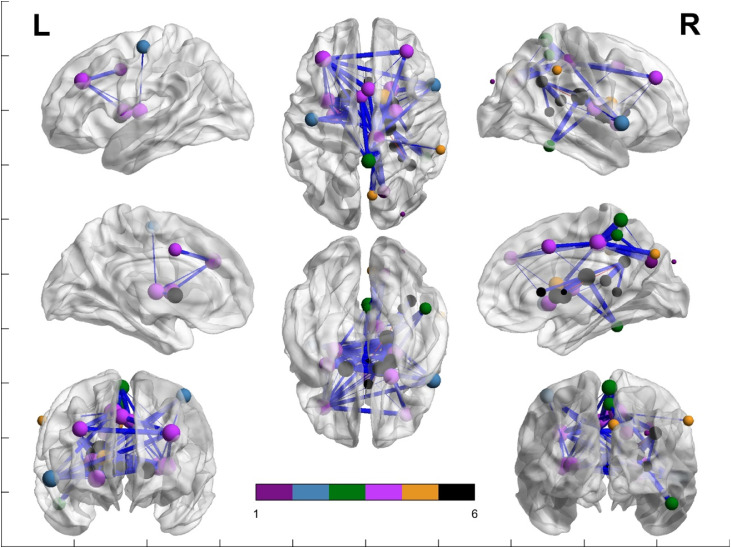
Spatial visualization of between-group differences in brain network organization.

*Note.* The visualization was generated using NBS-corrected results (Zalesky et al., 2010) (edge-level *p* < 0.001, component-level *p* < 0.05) in DPABINet and visualized with BrainNet Viewer (Xia et al., 2013). Colors represent the Yeo et al. (2011) networks, following their legend. Dark purple represents the ROIs included in the VN; blue represents the ROIs included in the SMN; green represents the ROIs included in the DAN; magenta represents the ROIs included in the VAN; orange represents the ROIs included in the FPN; and black represents the ROIs not belonging to Yeo’s network. Blue lines show areas that have significantly increased connectivity in adolescents compared to children. Red lines show areas that have increased connectivity in children compared with adolescents. The thickness of the lines represents the magnitude of the correlation coefficient (*r*) between the connected nodes. Node size reflects the sum of FC weights of each node.

Fig. 2 depicts the edge plot matrix, Fig. 3 shows the spatial connectogram and Fig. 4 provides the brain network representation.

## Discussion

This study is the first to integrate voxel-wise centrality metrics, DC-derived seed-based analyses, and network-based (edge-wise) connectivity to characterize functional brain organization in a large sample of typically developing children and adolescents. Across analytical levels, adolescents showed increased FC centered on thalamic and basal ganglia hubs (thalamus, putamen, pallidum, caudate), alongside cortical regions including precuneus, cuneus, and middle cingulate cortex, extending into VAN and FPN. Children showed higher FC in right angular gyrus, right superior occipital gyrus, and right thalamus, indicating a functional organization anchored in sensory-perceptual systems. In the present study, we operationally define 'integration' as the increased presence of functional connectivity edges across distinct large-scale networks. Overall, findings support a reorganization from sensory-based hubs in childhood toward integrated fronto-subcortical and attention-control architectures in adolescence.

Voxel-wise DC revealed complementary developmental profiles. Children showed higher DC in right angular gyrus and right superior occipital gyrus, consistent with a posterior-to-anterior maturational gradient in which sensory networks mature earlier than association systems. Developmental evidence indicates early maturity in visual/parietal intrinsic connectivity in childhood, followed by reorganization toward distributed configurations in adolescence (Dong et al., 2021; Xia et al., 2022). Posterior parietal regions such as angular gyrus function as early-developing hubs for default mode and perceptual-semantic processing (Fair et al., 2008; Seghier, 2013), while occipital regions show robust early FC with later refinement rather than increasing integration (Gao et al., 2015).

Adolescents showed increased DC in right thalamus and right middle cingulate cortex, key nodes for attentional control, performance monitoring, and cross-network integration. The thalamus is a central integrative hub coordinating communication across cortical networks (Hwang et al., 2017, 2021). The middle cingulate cortex, a core node of cingulo-opercular/salience systems, supports sustained attention and control-state coordination and continues to mature through late childhood and adolescence (Dosenbach et al., 2006; Kelly et al., 2008; Silveira et al., 2021). Together, higher DC in these hubs fits a shift from sensory-dominated organization toward coordinated cross-network architecture in adolescence. Together, DC findings indicate a gradual transition from reliance on posterior sensory systems toward maturation of integrative subcortical and cingulo-frontal nodes supporting goal-directed behavior.

Seed-based analyses showed coherent increases in adolescent FC across subcortical and cortical hubs. Adolescents exhibited strengthened connectivity of putamen, pallidum, and caudate, indicating greater basal ganglia integration (Greene et al., 2014; Leisman et al., 2014). These changes align with models proposing intensified dopaminergic modulation in adolescence supporting reward and motivational behavior (Luciana et al., 2012; Parr et al., 2021) and with evidence of increased centrality/connectivity of fronto-subcortical systems in this transition (Tian et al., 2023: Xu et al., 2021).

Beyond subcortical hubs, adolescents showed higher FC in frontal, parietal, and temporal association cortices, consistent with prolonged maturation of executive control, cognitive flexibility, and social-cognitive functions (Goddings et al., 2021). Adolescents also showed strengthened connectivity with posterior medial cortices (precuneus, cuneus), supporting internally oriented cognition and visuospatial integration (van Buuren et al., 2022). Increased coupling between these posterior hubs and thalamic/frontal regions may reflect growing integration among intrinsic, attentional, and sensorimotor systems, consistent with trajectories toward distributed large-scale coordination during adolescence (Cao et al., 2016; Wierenga et al., 2016). Seed-based results converge with prior evidence of increased adolescent connectivity in fronto-subcortical and association systems (Tian et al., 2023; Xu et al., 2021) and show that these differences are captured with DC-derived data-driven seeds. Overall, adolescence is characterized by strengthened coordination between subcortical regions and fronto-parietal systems supporting cognitive control, emotional regulation, and decision-making.

Network-level analyses indicated systematic increases in adolescent FC, with the strongest effects within and toward the VAN, FPN and SMN. The VAN emerged as a major integrative hub in adolescence (Dong et al., 2024), showing widespread strengthening with SMN, VN, DAN, and FPN. Given its role in salience detection and cognitive control (Farrant & Uddin, 2015; Wang et al., 2021), this pattern suggests substantial reorganization of salience-driven networks linking sensory input with subcortical and frontoparietal systems. The VAN also showed increased intra-network FC, consistent with evidence linking maturational changes to within-network connectivity and heightened DC (Dong et al., 2024) and with cortical refinement models (Casey et al., 2019; Tooley, Bassett et al., 2022). Clinically, VAN-subcortical FC predicts inhibitory control and substance use risk (Rømer-Thomesen et al., 2018; Wang et al., 2021) and relates to depression/anxiety profiles (Sylvester et al., 2013).

A key network-level feature was increased subcortical–cortical FC in adolescence. Basal ganglia nodes (bilateral pallidum, right caudate) showed widespread increases with cortical regions, especially VAN, SMN, and FPN, consistent with developmental reorganization of striatal connectivity (Padmanabhan et al., 2013). The right thalamus showed increased coupling with VAN, SMN, FPN, and DAN, consistent with its integrative role (Hwang et al., 2017). The hippocampus showed increased FC with putamen and right angular gyrus, aligning with evidence that hippocampal FC increases in adolescence with parietal/frontal memory networks (Warren et al., 2021), and suggesting broader multimodal integration. Longitudinal evidence indicates subcortical–cortical trajectories are connection-specific rather than uniformly increasing (van Duijvenvoorde et al., 2019). The prominence of subcortical hubs supports views that thalamus and basal ganglia are core transdiagnostic network components undergoing substantial reorganization in development (Choi et al., 2020).

The FPN showed a complementary pattern of increased adolescent coupling, mainly with VAN nodes (bilateral putamen, right pallidum) and with subcortical structures (right thalamus, pallidum). This suggests a developmental shift in which FPN becomes more integrated with salience and reorienting circuitry, consistent with FPN’s role as a flexible hub for cognitive control and goal-driven behavior (Marek & Dosenbach, 2018). FPN integration relates to cognitive ability (Sheffield et al., 2015) and fluid intelligence (Cole et al., 2015), which may become increasingly relevant with age. Prior work indicates FPN maturation involves increased connectivity (Chai et al., 2017), and disruption of this network is implicated across psychopathology (Marek & Dosenbach, 2018).

The SMN showed increased integration with salience/subcortical nodes in adolescence. Left precentral gyrus and right superior temporal pole exhibited robust FC increases with VAN-related subcortical structures. The left precentral gyrus strengthened connectivity with pallidum, thalamus, and putamen, consistent with maturation of automatization and motor control (Sydnor et al., 2023). The right superior temporal pole showed enhanced coupling with thalamus and pallidum, and with right angular gyrus and precuneus, possibly reflecting integration of sensorimotor and higher-order association functions (Murphy et al., 2018). SMN alterations are reported in ADHD (Chen, Lidstone et al., 2021) and OCD (Petrie et al., 2024); notably, somatomotor–putamen connectivity is linked to OCD symptoms moderated by stress (Petrie et al., 2024). In autism, disrupted age-related SMN pathways have been reported (Feldman et al., 2025).

The DAN showed selective strengthening in adolescence, particularly involving right precuneus and right inferior temporal gyrus, with enhanced integration with VAN, VN, and unclassified association areas. Given DAN’s role in top-down attentional allocation (Rohr et al., 2018), this pattern suggests refinement supporting flexible adjustment of attentional states (Gabard-Durnam et al., 2016). Increased coupling between precuneus and VAN nodes may indicate enhanced communication between internally guided attention and salience detection (Erb et al., 2022). This profile fits increasing cognitive flexibility and attentional switching demands, and aligns with developmental evidence (Schroeder et al., 2025; Westhoff et al., 2020).

The VN differences were characterized less by within-network change and more by strengthened cross-network communication. Right cuneus showed increased connectivity with DAN and VAN nodes, consistent with continued development of sensory–attentional coupling through adolescence (Sydnor et al., 2021). This suggests more efficient alignment between sensory processing and attentional selection, supporting increasing demands for complex visuospatial reasoning and socio-emotional functioning (Tooley, Park et al., 2022). Enhanced VN–DAN coupling may reflect a shift from stimulus-driven processing in childhood toward more goal-directed perceptual control in adolescence (Tooley, Park et al., 2022).

Interestingly, while the DMN is often highlighted in neurodevelopmental literature, our network-level analysis did not reveal significant changes across its core nodes. While the right precuneus, a primary DMN region, exhibited significant age-related increases in DC, its significant connectivity edges were directed toward the VAN and FPN. There is evidence suggesting that DMN reaches stability by late childhood, which may translate into focal reorganizations of key hubs rather than large-scale shifts in network-level connectivity (Jolles et al., 2011). Furthermore, inconsistent findings regarding DMN reorganization are not uncommon in the field, often reflecting differences in age ranges, sample sizes, and the specific connectivity metrics employed (Nomi & Uddin, 2015).

Several methodological considerations warrant mention. First, although multisite data introduce scanner/protocol variability, we mitigated this via harmonization and site covariates. Second, the cross-sectional design limits inference on within-subject trajectories, therefore, longitudinal work is needed. Third, analyses relied on DC as the only FC metric. Despite these limitations, this work provides a comprehensive multilevel characterization of normative functional maturation. The consistency of age effects across voxel-wise, seed-based, and network analyses suggests coordinated reorganization across scales rather than isolated changes. Using DC as a fully data-driven starting point identified developmentally relevant hubs without a priori ROIs, valuable in normative pediatric research where guidance for seed selection is limited. By deriving subsequent analyses from DC, we ensure that our findings are anchored in observed developmental differences rather than constrained by predefined models. These results are derived from the ABIDE I and II repositories, one of the largest and most established datasets in neurodevelopmental research. Rigorous QC, harmonization, and conservative thresholds strengthen interpretability. In the context of reproducibility concerns (Botvinik-Nezer & Wager, 2023), the use of public datasets and transparent pipelines provides a reproducible framework for future studies. Finally, further research should aim to replicate these analyses in other independent cohorts to confirm the robustness and generalizability of these maturational patterns across diverse populations.

## Conclusion

This study provides a comprehensive characterization of normative functional brain maturation by integrating voxel-wise DC, DC-derived seed-based connectivity, and network-level analysis in a large sample of typically developing children and adolescents. Across all analytical levels, adolescence was marked by strengthened FC within and between subcortical, attentional, and frontoparietal systems, particularly involving the thalamus, basal ganglia, VAN and FPN. In contrast, children showed greater reliance on posterior sensory and perceptual hubs, including the angular and occipital regions. Together, these findings delineate a developmental transition from sensory-anchored connectivity profiles in childhood toward increasingly distributed and integrative architectures during adolescence. This reorganization is consistent with the emergence of capacities for cognitive control, attentional flexibility, and goal-directed behavior.

Our connectivity findings contrast with previously reported activation patterns in children and adolescents (e.g., Tapia-Medina et al., 2025a). This apparent contradiction actually reflects a complementary pattern, highlighting a maturational shift toward greater neural efficiency. Accordingly, childhood is characterized by more diffuse and higher activation, whereas adolescence is characterized by progressively strengthened connectivity. This reflects a shift from less specialized processing to more specialized and integrated processing (Gozdas et al., 2018; Sanders et al., 2023). From this perspective, stronger FC in adolescents may indicate a more economical neural architecture.

By establishing age-specific patterns of local, regional, and large-scale functional organization, the present work represents an attempt to draw a normative reference framework for interpreting variability in neurodevelopment. For clinical and health psychology, establishing this baseline is crucial, as it offers a robust reference point to facilitate the early identification of atypical neurodevelopmental deviations associated with vulnerability and specific psychopathologies.

## Data availability

The data that support the findings of this study are openly available at http://fcon_1000.projects.nitrc.org/indi/abide/.

## Funding

This study was funded by Ministerio de Ciencia, Innovación y Universidades/Agencia Estatal de Investigación/10.13039/501100011033 and European Social Fund Plus (PRE2022-102574, Project CEX2021-001159-M-20-4), the Secretaría de Ciencia, Humanidades, Tecnología e Innovación (CVU867306), and the Agency for Management of University and Research Grants of the Catalan Government (2021SGR00366).

## Ethics statement

The studies involving humans were approved by the ABIDE I and ABIDE II projects that were carried out with approval from the local ethical committees at each participating site. The studies were conducted in accordance with the local legislation and institutional requirements.

## CRediT authorship contribution statement

**Merida Galilea Tapia-Medina:** Conceptualization, Methodology, Software, Data curation. **Raquel Cosío-Guirado:** Conceptualization, Methodology, Software, Data curation. **Maribel Peró-Cebollero:** Conceptualization, Supervision. **Cristina Cañete-Massé:** Conceptualization, Methodology, Supervision. **Erwin Rogelio Villuendas-González:** Conceptualization, Supervision. **Joan Guàrdia-Olmos:** Conceptualization, Supervision.

## Declaration of competing interest

The authors declare that they have no conflicts of interest.
